# Area of Habitat maps for the world’s terrestrial birds and mammals

**DOI:** 10.1038/s41597-022-01838-w

**Published:** 2022-12-03

**Authors:** Maria Lumbierres, Prabhat Raj Dahal, Carmen D. Soria, Moreno Di Marco, Stuart H. M. Butchart, Paul F. Donald, Carlo Rondinini

**Affiliations:** 1 Global Mammal Assessment Program, Department of Biology and Biotechnologies, Sapienza University of Rome, Viale dell’Università 32, 00185 Rome, Italy; 2 BirdLife International, David Attenborough Building, Pembroke Street, Cambridge, CB2 3QZ UK; 3 Department of Biology and Biotechnologies, Sapienza University of Rome, Viale dell’Università 32, 00185 Rome, Italy; 4 Department of Zoology, University of Cambridge, Downing Street, Cambridge, CB2 3EJ UK

**Keywords:** Conservation biology, Zoology, Biodiversity

## Abstract

Area of Habitat (AOH) is “the habitat available to a species, that is, habitat within its range”. It complements a geographic range map for a species by showing potential occupancy and reducing commission errors. AOH maps are produced by subtracting areas considered unsuitable for the species from their range map, using information on each species’ associations with habitat and elevation. We present AOH maps for 5,481 terrestrial mammal and 10,651 terrestrial bird species (including 1,816 migratory bird species for which we present separate maps for the resident, breeding and non-breeding areas). Our maps have a resolution of 100 m. On average, AOH covered 66 ± 28% of the range maps for mammals and 64 ± 27% for birds. The AOH maps were validated independently, following a novel two-step methodology: a modelling approach to identify outliers and a species-level approach based on point localities. We used AOH maps to produce global maps of the species richness of mammals, birds, globally threatened mammals and globally threatened birds.

## Abstract (summary)

**Table:** 

Measurement(s)	Species Distributions
Technology Type(s)	Area of Habitat
Factor Type(s)	Habitat
Sample Characteristic - Organism	Mammalia • Aves
Sample Characteristic - Environment	Terrestrial Habitats

## Background & Summary

Knowing the distribution of species is crucial for effective conservation action. However, accurate and high-resolution spatial data are only available for a limited number of species^1,2^. For mammals and birds, the most comprehensive and widely used global distribution dataset is the set of range maps compiled as part of the assessments for the International Union for Conservation of Nature (IUCN) Red List. These represent each species’ distributional limits and tend to minimize omission errors (i.e. false absences) at the expense of commission errors (i.e. false presences)^3,4^. Therefore, they often contain sizeable areas not regularly occupied by the species.

Maps of the Area of Habitat (AOH; previously known as Extent of Suitable Habitat, ESH) complement range maps by indicating potential occupancy within the range, thereby reducing commission errors^5^. AOH is defined as ‘the habitat available to a species, that is, habitat within its range’^5^. These models are produced by subtracting areas unsuitable for the species within their range, using information on each species’ associations with habitat and elevation^5–8^. Comprehensive sets of AOH maps have been produced in the past for mammals^6^ and amphibians^7^, as well as subsets of birds^8,9^. The percentage of a species’ range covered by the AOH varies depending on the methodology used to associate species to their habitats, and their habitats to land-cover, the coarseness of the range map, the region in which the species is distributed, and the species’ habitat specialization and elevation limits^5^. For example, Rondinini *et al*.^6^ found that, when considering elevation and land cover features for terrestrial mammals, the AOH comprised, on average, 55% of the range. Ficetola *et al*.^7^ obtained a similar percentage when analyzing amphibians (55% for forest species, 42% for open habitat species and 61% for habitat generalists). Beresford *et al*.^8^ found that AOH covered a mean of 27.6% of the range maps of 157 threatened African bird species. In 2019, Brooks *et al*.^5^ proposed a formal definition and standardized methodology to produce AOH, limiting the inputs to habitat preferences, elevation limits, and geographical range.

AOH production requires knowledge of which habitat types a species occurs in and their location within the range^1^. Information on habitat preference is documented for each species assessed in the IUCN Red List^10^, following the IUCN Habitats Classification Scheme^11^. However, the IUCN does not define habitat classes in a spatially explicit way, therefore, we used a recently published translation table that associates IUCN Habitat Classification Scheme classes with land cover classes^12^. Species’ elevation limits were also extracted from the IUCN Red List.

**Fig. 1:**
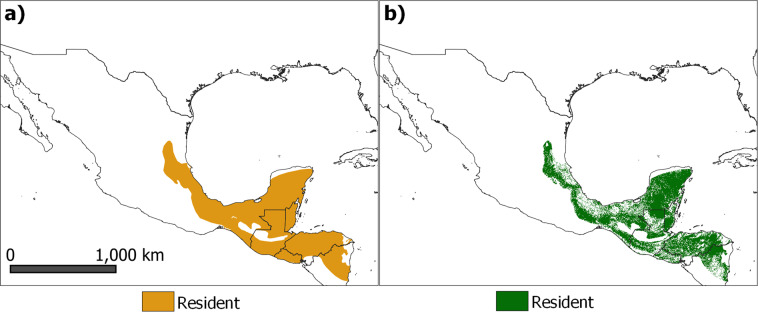
Spatial distribution maps of *Tangara abbas*. Maps represent (**a)** the geographic range^21^, and (**b)** the Area of Habitat (AOH) of the species. The AOH was produced by subtracting unsuitable habitats from the geographical range. This species’ habitats are forest and terrestrial artificial habitats and has elevation range of 0 – 1600 m.

**Fig. 2:**
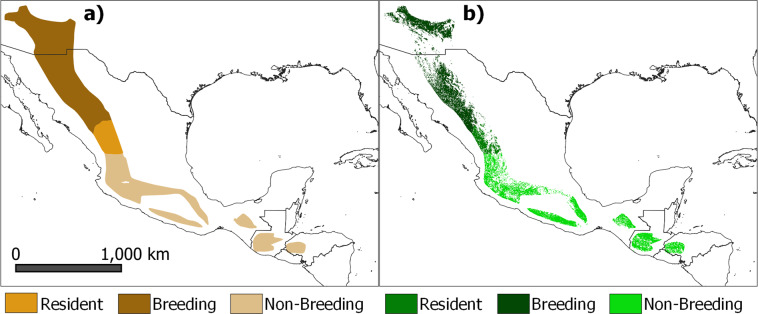
Spatial distribution maps of *Cardellina rubrifrons*, divided into resident, breeding and non-breeding areas for this migratory species. Maps represent (**a**) the geographic range^21^, and (**b**) the Area of Habitat (AOH) of the species. The AOH was produced by subtracting unsuitable habitats from the ranges. This species is a forest species with elevation rangelimits of 1500 – 3100 m.

**Fig. 3:**
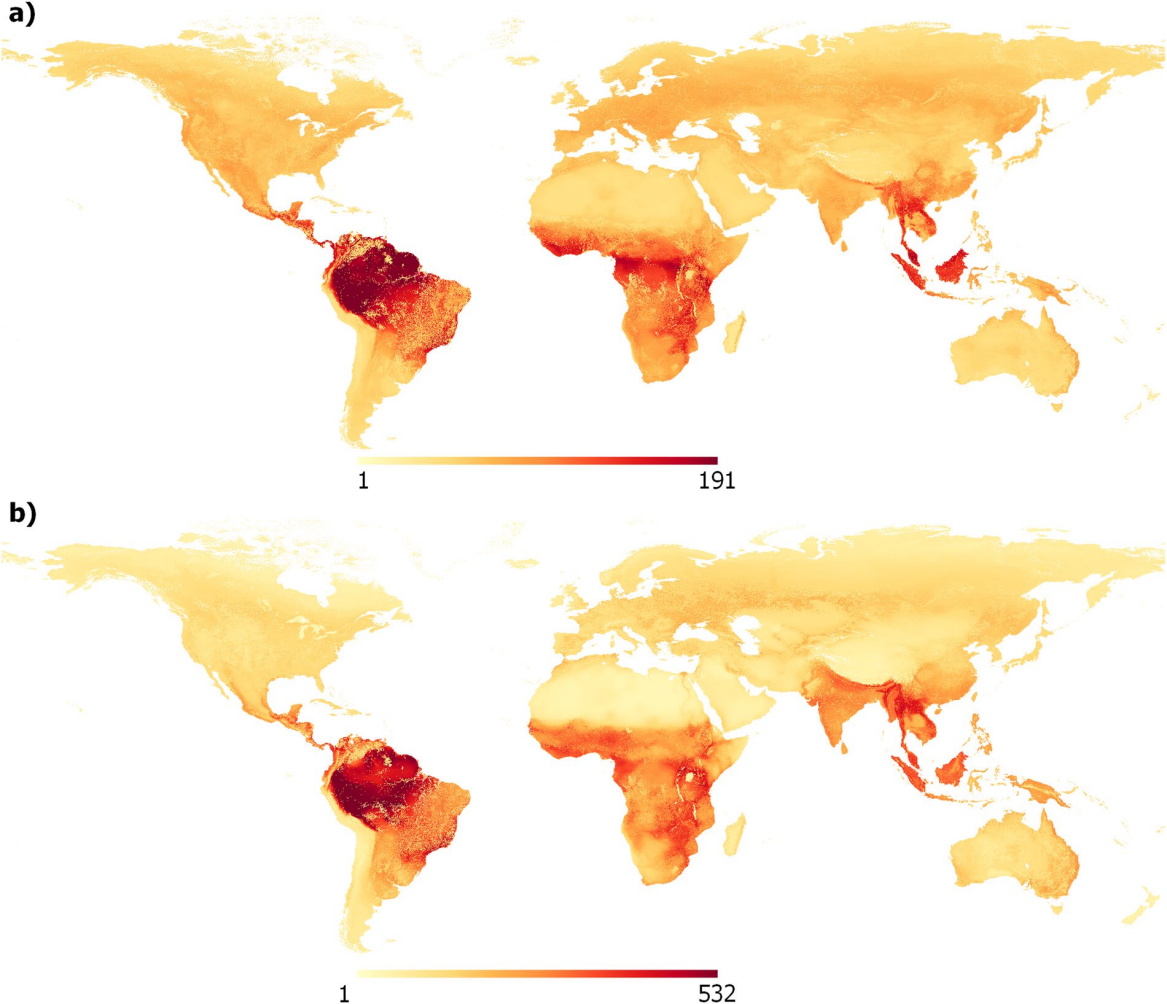
Global species richness maps for (**a**) terrestrial mammals (considering 5,481species) and (**b**) terrestrial birds (considering 10,651 species). Calculated by overlaying all species’ AOH per class, resulting inon the number of species at each grid cell, latitude/longitude grid at a resolution of 1°/1008 or approximately 100 m at the equator (EPSG:4326) with the ellipsoid WGS 1984.

We developed AOH maps for 5,481 terrestrial mammal species and 10,651 terrestrial bird species (Fig. 1). For 1,816 bird species defined by BirdLife International as migratory, we developed separate AOH maps, for the resident, breeding, and non-breeding ranges, according to the migratory distribution of the species (Fig. 2). The maps are presented in a regular latitude/longitude grid with an approximate 100 m resolution at the equator. On average, the AOH covers 66 ± 28% of the geographical range for mammals and 64 ± 27% for birds. We used the resulting AOH maps to produce four global species richness layers for: mammals, birds, globally threatened mammals and globally threatened birds^13^ (Fig. 3).

The AOH maps presented in this paper are more useful for some purposes than global species distribution models, as they reduce and standardize commissions^14^. They are especially useful for not well-known and wide-range species. However, we note that for well-known species alternative sources may have more accurate distributions^15^. Moreover, AOHs are affected by the bias and errors of the underlying data, especially relevant errors associated with documentation of species’ habitats and elevations, and the translation of habitats into land cover classes, given that habitat is a complex multidimensional concept that is challenging to match to land-cover classes^12^, and that the current version of the IUCN Habitat Classification Scheme on IUCN’s website is described as a draft version^11^.

The AOH maps have multiple conservation applications^5,16,17^, such as assessing species’ distributions and extinction risk, improving the accuracy of conservation planning, monitoring habitat loss and fragmentation, and guiding conservation actions. AOH has been proposed as an additional spatial metric to be documented in the Red List^5^, and is used for the identification of Key Biodiversity Areas^18^.

## Methods

We produced maps for extant species associated with at least one terrestrial habitat in the IUCN Habitat Classification Scheme^11^. We excluded a total of 342 species of mammals and 495 species of birds (6.2% and 4.6% out of 5,481 and 10,651 species, respectively). These comprised 135 mammals and 168 birds exclusively associated with marine habitats (i.e., marine neritic, marine oceanic, marine deep ocean floor, marine intertidal or marine coastal/supratidal), 29 mammals exclusively associated with caves and subterranean habitats, 131 mammals with no associated habitat codes, 8 mammals and 162 birds categorized as Extinct, 1 mammal and 5 birds categorized as Extinct in the Wild, 12 mammal and 142 birds that are restricted to small islands not included in the land-cover map we used, and 26 mammals and 18 birds that had null AOH, caused by errors in the coding of habitat and elevation^19^.

Species may have more than one range polygon, coded according to presence (the species is or was in the area), origin (why and how the species is in the area) and seasonality (seasonal presence of the species in the area)^20^. We used as a base for the AOH maps a predetermined subset of the IUCN Red List range^21^ polygons for each species^15^. Similarly to the Global Standard for the Identification of Key Biodiversity Areas Guidelines^22^, we selected range polygons with *extant* and *probably extant* presence; *native*, *reintroduced*, and *assisted colonization* origin; and *resident* seasonality for non-migratory species (all mammals and the 8,979 non-migratory birds). For migratory birds (1,816 species), we kept separated the ranges for *breeding* (1,446 species), *non-breeding* (1,550 species) and a combination of *resident* and *uncertain* (1,290 species) seasonality. We provide an R script to merge the AOH sub-maps into a single composite map for each species. We could not add separate AOH maps for migratory mammals to our dataset, as IUCN Red List provided insufficient data on migratory mammals’ range in different seasons^17^.

For 18 mammal and 22 bird species categorized as Critical Endangered, there were no presence polygons coded as *extant* or *probably extant*. To assist the conservation of these species, we produced AOH maps using the *possibly extinct* polygon for these taxa.

**Fig. 4:**
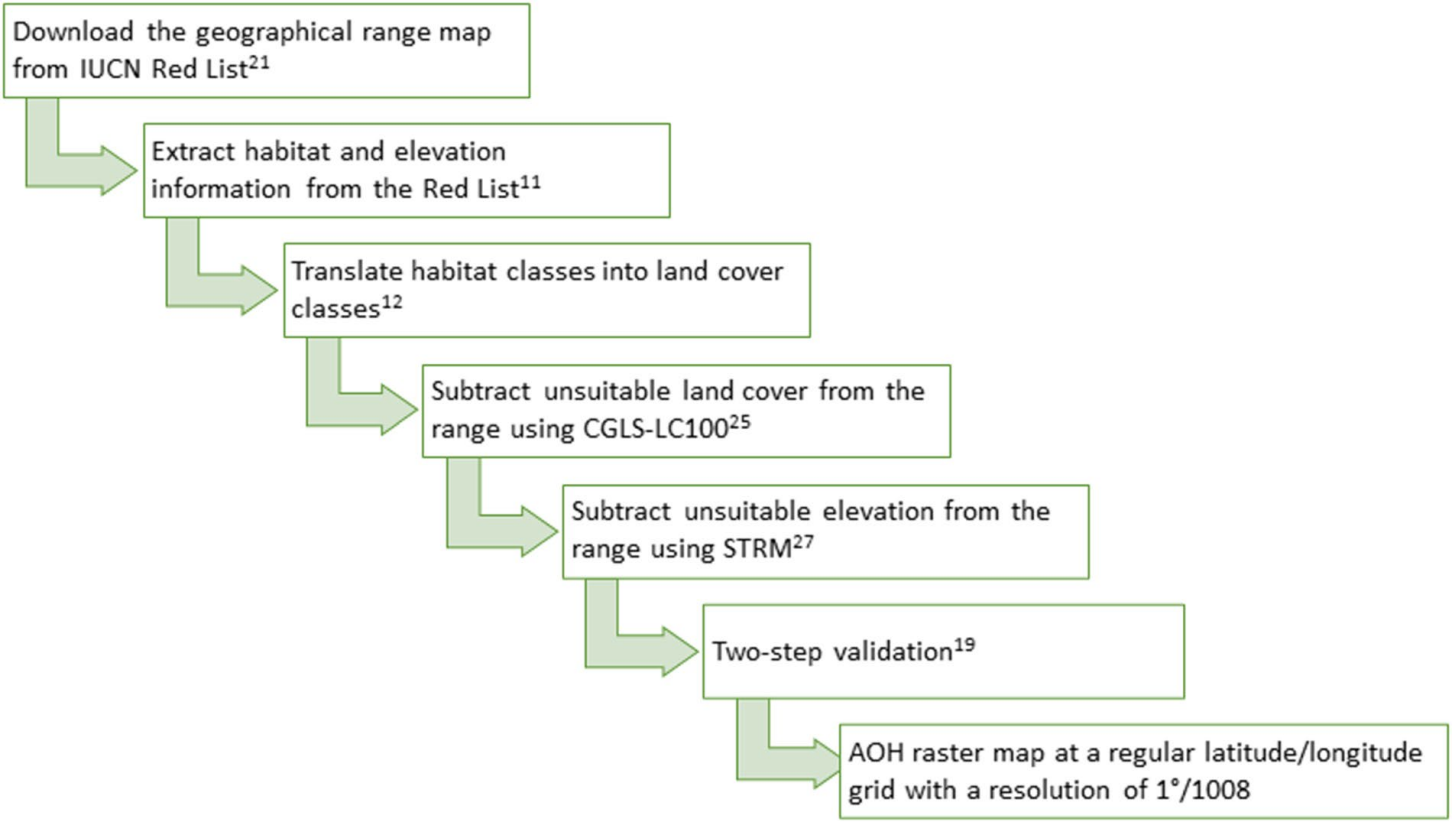
Step-by-step methodology to produce Area of Habitat maps (AOH).

AOH maps are produced by subtracting unsuitable areas from range maps, using data on each species’ associated habitat (Fig. 4). Habitat and elevation information was obtained from the IUCN Red list Version 2020-2^21^. As habitats in the IUCN Red List are not spatially explicit, although we note the existence of recently published maps^23^, we used a recently published translation table^12^ based on the Copernicus Global Land Service Land Cover (CGLS-LC100)^24,25^ and the European Space Agency Climate Change Initiative land cover 2015 (ESA-CCI)^26^. We developed the AOH maps based on CGLS-LC100 as CGLS-lC100 has a higher resolution and accuracy than ESA-CCI. CGLS-LC100 is in a regular latitude/longitude grid (EPSG:4326) with the ellipsoid WGS 1984 and a grid resolution of 1°/1008 or approximately 100 m at the equator, defining the resolution of the AOH maps. The translation table presented the relation between each habitat in the IUCN Classification Scheme and each land-cover class as a continuous variable. To create a binary table of association or non-association, Lumbierres *et al*.^12^ proposed three potential thresholds based on the tertiles of the positive association values of the table. We produced maps for the three proposed thresholds and evaluated the ratio of AOH area to range area. As the threshold increased, the ratio decreased, and the results were more similar to previous AOH maps^6^. Dahal *et al*.^19^ evaluated these three thresholds and corroborated that an increase in the threshold did not reduce the performance of the AOH maps during validation. Therefore, we present the maps produced using the highest threshold (odds ratio >1.7). Species’ elevation limits were extracted from the IUCN Red List^21^. To subtract the parts of the range outside the elevation limits, we used the Shuttle Radar Topography Mission (SRTM)^27^ map, resampled at the resolution of the CGLS-LC100 (Fig. 4).

One of the main complexities of this analysis was the large amount of data generated in the process. Therefore, the AOH maps were produced using the GRASS GIS^28^ software, which allows processing of large amounts of raster data efficiently. The AOH production procedure consisted of four steps, following Rondinini *et al*.^6^: (1) Transforming the habitat codes of each species into land cover classes using the translation table^12^. (2) Creating a base map that combines the information on land cover and elevation. (3) Creating reclassification files containing the information on land cover and elevation preferences for each species. (4) Reclassifying the base map based on the reclassification files to create the AOH for each species. Once the AOH were produced, we calculated richness maps by stacking the AOH maps, producing maps representing the number of species at each grid of 100 × 100 m. Migratory species’ AOH maps were merged before calculating richness to ensure each species counted once.

## Data Records

The AOH data, including tables and maps, are stored in the Dryad Open Access Repository 10.5061/dryad.02v6wwq48^29^. The data are organized by taxonomic Class with zipped folders by taxonomic Order. In the case of birds, we separated migratory species from non-migratory species. We added a folder containing the richness maps for each class of all species and of globally threatened species. In each folder, we included a table with information about the excluded species, indicating the reason for exclusion(Mammls_list_excluded.csv, Birds_list_excluded.csv); and a table with the included species, the model prevalence (AOH range ratio) and the results of the validation developed by Dahal *et al*.^19^ Mammals_list_AOH.csv, Birds_list_AOH.csv). For migratory birds, we included a table specifying which maps (breeding, non-breeding and resident) each species has (Birds_Migratory_list_AOH.csv) and code to merge the different parts of the AOH (merge_migratory_AOH_code.R).

## Technical Validation

The accuracy of the AOH maps was assessed using a novel methodology developed by Dahal *et al*.^19^ and full details of the validation are provided there. This methodology allowed validation of AOH maps for species with or without point localities. Previous AOH maps were validated only using point localities and polygons of occurrence^6–8^, leaving some of the AOH maps unvalidated.

Our method employed a two-step approach. The first step identified potential systematic errors in the AOH maps using a modelling approach. This approach flagged 178 and 64 AOH maps for birds and mammals respectively that were carefully studied to identify the sources of potential errors. These potential errors were caused by inaccuracies in species’ elevation limits, habitat coding or the translation table^12^ used to assign habitat to land cover. Work is currently underway to address these issues, and improved AOH maps will be available in the future for download at https://www.iucnredlist.org/resources/grid/spatial-data. A complete list of flagged maps can be found in Dahal *et al*.^19^.

The second step used point localities to validate the maps at the species level. Point data for mammals were downloaded from GBIF^30^ and for birds from eBIRD^31^. Dahal *et al*.^19^ applied several filters to ensure that only high-accuracy points were used for the validation. Only points with coordinate uncertainty lower than 300 m were retained for mammals and only stationary points were selected for birds as they have a coordinate uncertainty of less than 30 m. Also, it was ensured that points fell inside the mapped distribution and that at least 10 point were available for each species. A temporal filter of 2019–2020 was applied because the point localities from 2005–2018 were used to calibrate the habitat-land cover model by Lumbierres *et al*.^12^. This resulted in 4889 birds (46% of all bird species) and 420 mammals (8% of all mammal species) that had enough available point locality data for validation. For mammals, this represented 157 species more than in a previous set of AOH^6^ maps published in 2011.

To validate the AOH maps, the proportion of points localities falling inside the AOH (point prevalence) was compared with the AOH/range ratio (model prevalence). If point prevalence exceeded model prevalence, the AOH was assumed to be better than a random distribution within the species’ range^6^. We found that AOH maps were better than random for 95.9% bird and 95% of mammal species, among those with validation data. The unavailability of point locality data for half of bird species and most mammal species remains a major limitation of the validation analysis. However, the first step of the method allowed us to assess at least the general soundness of AOH maps for species that did not have suitable point localities for validation.

## Usage Notes

The maps are presented in raster byte GeoTIFF format. The values of the maps are 1 for the AOH area and Null for the background. The geographical extent of each map is defined by the species’ range. Each species map is presented separately with the species binomial name, and the genus and specific epithet separated by an underscore. For migratory birds we produced three different maps, that are coded using, R, B and N for resident, breeding and non-breeding AOH maps, respectively. We present code written in R to merge the different AOH maps for migratory species according to the needs of the user. For species with null AOH we recommend using the mapped range.

### Base map

The base map is the map that is reclassified to produce the AOH. Each cell value is a combination of land cover and elevation, where the three first digits represent land cover and the three last digits elevation in m/10.

### Reclassification Files

The GRASS reclass function has a specific format for the reclassification instructions. The script produces reclass files to apply to the base map in GRASS to produce maps of area of habitat for terrestrial species. It reads a file that contains land cover associations, with the following column headers: species name, one column per land cover class (with numeric column names for land cover; e.g., 10, 20, 210), and two columns representing elevation range (elevation_min and elevation_max). If the elevation range for a species is unrecorded, it is set to 0–9000 m.

### AOH production

AOH is confined inside the geographical range. The geographical range maps can be downloaded from https://www.iucnredlist.org for mammals, and http://datazone.birdlife.org/species/requestdis for birds. The ranges are imported into GRASS and rasterized. The ranges are used to mask the area outside the species distribution. Inside the non-masked areas, the base map is reclassified using the reclassification file.

## Data Availability

The code to produce the AOH is derived from code produced by Rondinini *et al*.^6^. AOH maps are produced reclassifying a base map that contains information on elevation and land cover. The geographical range maps are used to mask the areas outside the distribution of the species. Each species has a reclassification file that indicates which land cover classes and elevations are suitable. To transform the habitat information into land cover we used the translation table^12^. The code is both in GRASS and R.
